# Kick-starting concept formation with intrinsically motivated learning: the grounding by competence acquisition hypothesis

**DOI:** 10.1098/rstb.2021.0370

**Published:** 2023-02-13

**Authors:** Francesco Mannella, Luca Tummolini

**Affiliations:** ^1^ Institute of Cognitive Sciences and Technologies, CNR, 00185, Rome, Italy; ^2^ Institute for Future Studies, IFFS, Box 591, 101 31, Stockholm, Sweden

**Keywords:** concept formation, grounded cognition, success semantics, intrinsically motivated learning, sensorimotor contingencies, manifold alignment

## Abstract

Although the spontaneous origins of concepts from interaction is often given for granted, how the process can start without a fully developed sensorimotor representation system has not been sufficiently explored. Here, we offer a new hypothesis for a mechanism supporting concept formation while learning to perceive and act intentionally. We specify an architecture in which multi-modal sensory patterns are mapped in the same lower-dimensional representation space. The motor repertoire is also represented in the same space via topological mapping. We posit that the acquisition of these mappings can be mutually constrained by maximizing the convergence between sensory and motor representations during online interaction. This learning signal reflects an intrinsic motivation of competence acquisition. We propose that topological alignment via competence acquisition eventually results in a sensorimotor representation system. To assess the consistency of this hypothesis, we develop a computational model and test it in an object manipulation task. Results show that such an intrinsically motivated learning process can create a cross-modal categorization system with semantic content, which supports perception and intentional action selection, which has the resources to re-enact its own multi-modal experiences, and, on this basis, to kick-start the formation of concepts grounded in the external environment.

This article is part of the theme issue ‘Concepts in interaction: social engagement and inner experiences’.

## 1. The kick-starting problem: how to get cognition off the ground

Infants are able to form concepts and other mental representations while learning to interact with their physical environment, with their carers, and with their peers. Although it has been found incredible on logical grounds by some [1], most of us—scientists as well as laypeople—share the intuition that newborns are capable of developing a meaningful understanding of their situation out of the ‘blooming, buzzing confusion’ [2, vol. 1, p. 488] that their initial sensory and motor contact with the outside world must be.

Consider the interaction between the 4-month-old infant and the rattle as recounted by Piaget in his seminal study [3]. At this age, the infant has partial control over her eye and hand movements. However, by exploring the rattle, the infant may accidentally create visual or auditory effects, which will induce further movements and create repeated experiences until the infant learns to shake the rattle intentionally (see, for instance [4–6]). As a further consequence of learning to act intentionally, a crude but meaningful understanding of what a rattle is may also form, perhaps something that can be described as ‘a thing I can grasp and shake and make noises with’ [7, p. 137]. However, what mechanism can support the formation of meaningful conceptual representations starting from these initially accidental encounters? How can meaning emerge from something that is meaningless to begin with? Or, to put it differently, how do physical processes internal to the organism get semantic content in the sense of being connected to the outside world and be *about* it?

Despite its undeniable philosophical flavour, the underlying origin problem is relevant for developmental psychology as well as for cognitive science as a whole given the peculiar explanatory significance of representational content in empirical practice [8].

In this paper, we advance a novel hypothesis for a mechanism that can solve the kick-starting problem and can support concept formation starting from spontaneous interaction with the environment. To assess its consistency and provide a proof-of-principle, we formalize the hypothesis in a computationally specified process model and test it in a manipulation task. In what follows, we first present the frameworks of grounded cognition and the theory of event coding within which our proposal is placed and then discuss some of their limitations. Next, we introduce our hypothesis, the cognitive architecture that formalizes it, and its implementation in a computational model. Finally, we present some simulation results showing how conceptual representations can develop starting from an initially meaningless sensorimotor stream of data. We conclude by discussing several implications of this proposal including its psychological and biological plausibility.

### (a) Grounding representations by perception is insufficient

According to a popular approach, a crucial way in which concepts and other high-level cognitive functions derive their meaning is by remaining tied to their experiential origins. In this view, concepts derive their semantic content by being *grounded* in perception, action and introspection [9], with ‘grounding’ being defined as the process through which representations are connected to what they are about, the capacity to autonomously link them with referents in the outside world [10]. More specifically, according to the framework of grounded cognition [11], conceptual knowledge of a category (e.g. rattle) is made possible by the re-enactment of a multimodal representation system integrating the perceptual, motor, and introspective states acquired during experience with the world, body and the mind itself. In this view, simulating the modal experiences derived from online interaction with the outside environment enables the possibility of offline re-use of these multimodal representations in other cognitive processes and actions. Importantly, however, given these premises, what is endowed with meaning or semantic content ultimately are the multimodal representations themselves, and the meaning acquired by conceptual representations is derivative of the perceptual ones. Therefore, to clarify how to ‘kick-start’ the formation of meaningful concepts, one needs to explain how these multimodal representations get their meaning in the first place, how sensorimotor representations become themselves grounded in the environment.

A main assumption, which is still shared by many grounded approaches, is that multimodal experiences are the product of a statistical learning process that is dominated by the sensory input. Statistical learning is based on a feed-forward flow of information from multimodal sensory inputs towards higher levels in the processing hierarchy in which these bottom-up signals are eventually integrated [12–15]. Inspired by the Bayesian brain hypothesis [16], more recent versions of this view have highlighted the fundamental generative nature of perceptual processes, which do not only rest on bottom-up, multimodal flows but also on their integration with top-down predictions, enabling the possibility to infer that a certain (perceptual) hypothesis is true given the current sensory pattern (as in predictive coding, see [17,18]). Since in the generative approach, this process can naturally be inverted and used to infer the sensory or motor states one would experience given some (hidden) state of the world, this approach may also account for the process of re-enacting multimodal experiences as assumed by the grounded cognition framework. However, despite their differences, these views still rely on the same grounding process, which is based on the causal connection established when the outer world of physical events (or the inner world of motor and introspective states) impinges on a perceiving subject. In this approach, grounding is in the end a *passive* process. Boosted by the deep learning revolution in artificial intelligence [19], the passive approach entailed by statistical learning has indeed obtained impressive results by solving very challenging tasks in computer vision and natural language processing as well as by combining the two in the so-called ‘grounded’ language models [20]. Still, it has been argued that these computational feats fall short of breaking into the ‘barrier of meaning’ [21]. An important reason for this limit is that statistical learning in the form of passive exposure to a stream of sensory inputs creates representations of sensory data in the form of internal ‘encodings’, whose information can be decoded only by someone who already knows what is correlated with what [22]. While these representations are meaningful from the perspective of a third-party observer who can access the particular features in the external environment to which biological or artificial neurons respond, they cannot in fact be decoded from the first-person perspective of a learning system who cannot rely on such direct access (for a more extended argument see [23–25]).

### (b) Grounding representations by action is unsuccessful

In light of these concerns, an alternative view of grounding as an *active* process has emerged [7,25–28]. Somewhat less intuitively, here the idea is that the internal representations of an organism are connected to the environment not (primarily) via its sensors but through its effectors, or better, by detecting the (predictable) changes that the organism’s actions have on its sensory inputs and by learning to master the motor-sensory contingencies [29] required for skilled online situated interaction. In this action-based approach, establishing a causal connection between the environment and the agent’s sensory inputs is not a sufficient grounding process, but being able to anticipate the sensory consequences of one’s own actions is necessary too. Grounding should be understood as a *causal-predictive cycle*, an interactive process of predictive control and causal feedback [26], which is made possible thanks to the acquisition of internal models in the brain. These models are conceived as information structures mimicking and supporting the sensorimotor loop that is responsible for the adaptive online agent’s interactions with the environment [26,28,30,31]. In this view, the crucial explanatory role is played by prediction errors indicating the match/mismatch between top-down predictions of the sensory consequences of actions and the actual bottom-up sensations arising from online interaction with the environment. Grounding is assured by a continuous, direct process of ‘verification’ and is threatened by a failure to correctly predict what happens given that one has acted.

Consider, for instance, how the concept of ‘rattle’ might be grounded in the action-based approach. Knowledge of the concept type amounts to acquiring an internal (generative) model encoding the sensory consequences that would be experienced were one to act on the object. Acting on the basis of the internal model, e.g. preparing to grasp a rattle on the belief that a rattle is available in the environment, puts this knowledge to the test because successfully carrying out the action reveals that the beliefs on which the action is based are correct. Among other things, the successful execution of the action of grasping a rattle presupposes the actual presence of the rattle. Being able to predict with respect to action—its outcomes—provides an operational definition of interpretation from the point of view of the agent herself, who, in this way, has the possibility to verify her knowledge by comparing predictions to actual sensations.

The action-based approach to grounding is consistent with the pragmatist approach to mental content [32]. In this approach, the content of a mental representation is in fact determined (or fixed) by the effects that the mental representation has on behaviour rather than by what has caused it. This is so, because getting what one wants (success or goal fulfilment) depends on whether the way one represents things to be is the way they actually are. In the formulation provided by *success semantics* [33,34], the conditions determining what is represented by a mental representation (its content) are the conditions in which the action guided by the representation is successful, its success conditions. Still, even if the action-based approach offers a way to understand how the mental representation of an object can be meaningful—in the sense of being connected with what it represents—it does so by assuming that the agent already relies on a well-formed action repertoire. However, since an action cannot be reduced to a mere bodily movement or a sequence of motor commands but also requires a reference to the outcome or effect towards which the bodily movement is oriented and that triggers it—its goal [35,36], *actions themselves have meaning* [37,38]. Indeed, without such connection with a goal, the notion of success would be difficult to comprehend. Thus, the content of internal ‘belief-like’ representations is determined with reference to other ‘goal-like’ representations which are semantic nonetheless. However, how do actions, the goal-directed movements needed to ensure grounding to mental representations, get their meaning as well?

Without an account of how an agent can learn to perceive and act that does not smuggle further representational notions along the way, the action-based view of grounding cannot solve the kick-starting problem after all.

### (c) Grounding representations by competence acquisition: concept formation while learning to perceive and act

A theoretical framework that addresses the origin of action as goal-directed behaviour and emphasizes its intimate connection with perception is the Theory of Event Coding (TEC) [39]. According to TEC, perception and action are not distinct processes that need to interact with each other, but should instead be conceived as a single process: the carrying out of a movement to generate a particular sensory event. Such process is construed as ‘perception’ when the focus is on the sensory event that is generated but it is viewed as an ‘action’ when attention is centred on the process that generates such event. According to TEC, the integration of perception and action is possible because perceptual events and action effects are coded in the same representation space and are thus commensurable (common coding [37]). Learning to control an action intentionally—the acquisition of action control—rests on the ability to detect and encode the contingencies between self-performed movements and sensory effects. TEC assumes that these links are the product of a spontaneous associative (Hebbian) action-effect learning mechanism, which discovers and creates bidirectional associations between a motor pattern and its sensory effects, and encode them in a common representation space [40]. As a consequence, independently of whether a perceptual event is activated by a stimulus or is endogenously generated (a goal), it will also lead to the selection of the motor programme needed to generate it [41,42].

Unfortunately, despite the merits of this general framework, the incidental detection of correlations between motor programmes and sensory events in a high-dimensional sensory and motor stream of data characterizing, for instance, the infant’s first explorations of its rattle, is an intractable problem. Given the redundancy of the motor system [43], to detect correlations in these continuous streams one needs to deal with infinite sequences of motor commands and trajectories that lead to the same effects in the absence of a univocal relationship between them.

This problem can be solved, and a unique common lower-dimensional manifold between the sensory and motor spaces can be found, if instead a reward signal is used as a filtering mechanism to select the relevant sensory and motor events from which to learn. Importantly, this signal does not need to consist of a standard extrinsic reward signal, which, for many skills, is sparse (many correct actions are often needed before getting the reward) or even completely absent (what is the extrinsic reward of learning to grasp the rattle and where does it come from? [44]), but it may as well be generated by a motivation that is *intrinsic* [45–47]. Intrinsic motivations are motivations that produce learning signals, or trigger the performance of exploratory behaviours, to drive the acquisition of knowledge and skills without a clear instrumental or strategic value in the present context even if there might be one in the future. Their role in learning and development is nowadays increasingly recognized [48,49]. Various intrinsic motivations have been distinguished [50], some tied to the novelty or surprise of perceptual events [51], and others based on an agent’s competence, her capacity to successfully accomplish a desired outcome [52]. Adopting often overlapping constructs [53], it has been shown that motivations related to acquisition of competence and control over the external environment are crucial for human well-being and health outcomes [54]. In the developmental context, these competence-based motivations have been assumed to play a crucial role for motor-skill development in infants [55], and computational work in machine learning and robotics have indeed demonstrated that they can be used to directly guide the autonomous learning of skills [56], and also of actions and goals [57,58].

Importantly, beside the acquisition of motor skills, competence-based intrinsic motivations can also be effective for acquiring new knowledge. For instance, theoretical work [59,60] has shown that an agent, whose learning and exploration is guided by an intrinsic motivation for competence acquisition, can discover and form representations of the possible sensory events that it can cause when touching its own body and become able to select and pursue them as goals. Importantly, in addition to acquiring the ability to touch its body in a goal-directed way, the agent ends up acquiring a representation of its own body too, its own body schema.

Here, we extend this perspective to the formation of object concepts.

#### (i) The grounding by competence acquisition hypothesis

Adopting a geometrical perspective [61], we conjecture that to kick-start concept formation, the control system of an organism needs to be endowed with a common lower-dimensional representation space in which the distance between representations of sensory inputs and motor programmes can be computed even when points in this internal space are meaningless at first. Leveraging on this internal space, we posit that by acting and observing how (multimodal) sensory representations change in this internal space, the topologies in which motor and sensory inputs are represented become aligned with each other over time. This topological alignment entails that the lower-dimensional projections from each modality converge in a point of the internal space that represents a single sensorimotor contingency occurring in the world. We propose that an appropriate topological alignment can be achieved if learning is guided by a signal measuring the convergence in the internal space between the internal representations of a motor programme and of sensory events. Since this signal is influenced by the contingency between action and sensory changes, and it is entirely derived in the internal space itself, it is interpreted as a signal of *competence*: of the agent’s ability to generate an effect in the environment. By minimizing the distance between motor and sensory representations in the internal space, and thus maximizing the agent’s competence, the internal signal guides learning and plays the role of an intrinsic motivation. The intrinsically motivated learning process results in the acquisition of representations that are sensorimotor in nature, are grounded in the environment (distal reference), and are amenable for offline re-enactment and use by other cognitive processes (concept formation).

## 2. The cognitive architecture

Our main hypothesis is that meaningful sensorimotor representations can be spontaneously acquired by using an internal signal derived from the distance between motor and sensory representations. In order to align the motor and sensory spaces, the mappings from the sensory and motor modalities to the internal representational space need to be dependent on each other during learning so that they end up being represented in what is in fact the same, *common* space. Crucially, to obtain such alignment, the motor space must be defined at a correct level of abstraction. In particular, the motor space cannot be identified with the space of motor commands, defined as the inputs to the motor actuators at a single instant relative to the current sensory state. Indeed, it must be possible to relate a group or sequences of motor commands—a motor programme—to a single sensory representation to allow for the generalization of sensorimotor representations, which should be independent from the current state of the motor actuators. Thus, the right level of abstraction of the motor space can be found at the level of policies, which are *functions* mapping sensory states to motor commands. Such policies can be implemented for instance as the set of weights (parameters) of a neural network taking as input the current sensory states and giving as output commands to the motor actuators (figure 2*c*). Given this level of abstraction, the relevant motor space is constituted by the space of the policy parameters that define all possible policies. For this reason, in what follows we will use the expression policy space to refer to the space of the motor programmes defining the behavioural repertoire of the agent.

We propose that perceptual and action representations thus emerge by aligning the representational space of motor programmes (policy parameters) with the representational space of different sensory modalities (sensory states), so that a point in this common space ends up representing both a multimodal sensory state and the motor programme that would generate it (a policy of behaviour). As assumed in the TEC [39], perception and action turn out to be one and the same integrated process.

With these requirements in mind, two main control layers of the resulting architecture can be identified: (i) an *offline control system* for action selection, which is able to generate policies and sensory state predictions starting from activities at the level of the common space of representations, together with a *learning process* by which sensory and motor mappings become aligned into this common representational space; and (ii) an *online control system* that uses the current policy as a mapping between sensory states and motor commands so that the agent can resolve the closed-loop interactions in a given context and it is able to reach the predicted states (i.e. the sensory states that are aligned with that policy).

In what follows, we first discuss the role of the offline control system for action selection and discuss how it can be implemented. We describe the learning process by which a common internal space in the offline control system can be generated on the basis of distances between internal representations in a bootstrap manner. We briefly present the general algorithm for the implementation of the topological mappings that we use and the algorithm developed for their supervised updating based on distances between representations. Next, we describe the online control system, which is used to specify the behaviour of the agent in the actual context. Finally, we present a computational model implementing the two-layer architecture in a manipulation task and the simulation setting to which we refer in the rest of the paper.

### (a) The offline control system: alignment of multi-modal topologies for perception and action

The main role of the offline control system is to select the policy that will be executed by the online controller in the actual environment [62]. As in TEC, two alternative modes should be distinguished: an exogenous or stimulus-tied mode driven by the environment as well as an endogenous goal-driven mode of action selection, defined by reference to a self-generated process potentially decoupled by online interaction in a given context [41]. Previous research has argued that both modes are important to understand the sensorimotor origins of higher cognition [63,64]. These two modes of action control become available when motor programmes (policies) are integrated with their sensory effects in the internal representation space. In particular, an internally generated process might in principle select the relevant policy by directly accessing its representation in the internal space. At the same time, however, actions can also be selected exogenously, by mapping an internal representation of the current sensory state backwards to the corresponding policy pattern.

To achieve this integration between perception and action, the offline control system implemented here is equipped with a mechanism for reducing the dimensionality of input patterns in the sensory and motor modalities. This mechanism has two important features. First, it maintains the information about the topology of each input space by finding a lower-dimensional manifold within the original space of each sensory and motor modality. Second, it aligns these manifolds in the internal representation space. As a result, each point in the representation space becomes a multimodal representation of a sensorimotor event. Representations can then be activated by selecting a point in the internal space such that points which are closer in this space represent sensorimotor events that are more similar to each other.

#### (i) An intrinsic signal to detect motor-sensory contingencies

**Figure 1.**
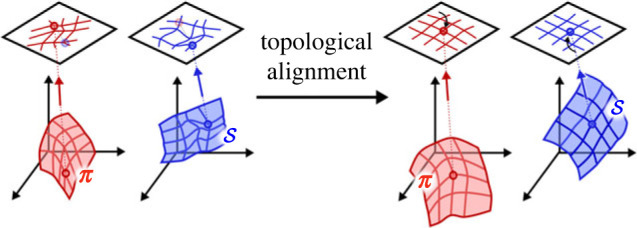
The diagram illustrates how the distance between two representations in the common internal space can be exploited to align two topological maps. In the visual example, input patterns from a policy ***π*** and a sensory space ***s*** are represented in an internal lower-dimensional space. The lower-dimensional manifolds to which each modality space is reduced are initially random and uncorrelated. Topological alignment shapes both manifolds so that a point in the internal space maps to points in the space of each modality that should reflect a motor-sensory contingency. (Online version in colour.)

A learning process is needed for the alignment of the emerging sensory and policy topologies within the common lower-dimensional representation space. Thanks to this process, representations of those features that are relevant to the representation of the current policy are positioned in the same region within each manifold. For this learning mechanism to be implemented two requirements must be fulfilled. First, the lower-dimensional projections of each modality must share an intrinsic internal metrics so that regions in the space of one modality can be related to regions in the space of another modality. Second, a function must be found to relate patterns in different modalities with each other so that sensory inputs that are associated with a specific motor programme are represented in the same region of the internal space. The two requirements can be merged by assuming that the projections of all modalities lie in the same lower-dimensional inner space and, consequently, by observing that the relationship between motor programmes and sensory inputs can be detected *through* a measure of the distance between their projections in this inner space. In particular, we propose that learning should be dependent on the amount of *decrease in the distance or convergence* between representations in the common space (figure 1). The assumption we make in using such a measure of convergence is that *when representations of sensory inputs in the internal space get closer to the representation of the policy that is in fact controlling the agent’s behaviour, there is a higher chance that the current policy is in fact causally responsible for those sensory inputs*. In other words, a candidate motor-sensory contingency or action effect is detected. Granted this, the convergence measure can be used as a learning signal for the development of topological mappings. In particular, this signal can be used as a filter to select for learning only those sensorimotor inputs for which there is convergence in their internal representations, so that the development of internal topologies is constrained. Thanks to this convergence process, representations of final sensory states and representations of policies eventually overlap and a representation of the motor-sensory contingency or action-effect is obtained.

Importantly, an agent who is able to learn on the basis of an internally derived signal selecting the sensory experiences influenced by its actions, also acquires the skill to generate those experiences by acting, and is in fact an agent who is intrinsically motivated to ‘interact effectively with its environment’, i.e. an agent whose learning is guided by an intrinsic motivation to acquire and increase its own competence [52].

#### (ii) The intrinsic signal bootstraps the learning of topologies

At the beginning of the agent’s learning, topologies are not yet developed. Thus activations in the internal space are only randomly related to the sensory inputs occurring to the agent. In the same way, the representations in the internal space from which the policies are generated are randomly related to the policies that are in fact executed by the agent. The agent thus starts with internal representations that are meaningless at first. Importantly, however, as long as subsequent sensory states are detected, despite the agent’s initial random behaviour (which here amounts to selecting a policy among those currently available) the internal representations of the different sensory modalities will move within the inner space. Over time, given the common metric of the internal space, these sensory representations happen to get closer to the internal representation which generated the current policy. These are the sensory inputs and motor programmes that will be assigned a higher amount of learning signal. The sensory inputs that are selected by the learning signal and the policy that was executed are then used as the current dataset for updating both sensory and motor topologies. Thus all topologies, including those representing motor programmes or policies, are updated so that they are more aligned in the region where representations converged. In sum. the same learning process guides the development of all topologies, using the fact that an initial proximity between two representations within initially randomly mapped internal spaces bootstraps the learning of both mappings.

Updates in the alignment of sensory and motor topologies thus correspond to the discovery and encoding in the common representational space of motor-sensory or action-effect contingencies. Indeed such an emerging representation in the internal space is related to a policy that is able to obtain a given outcome. This outcome is the state in which those sensory patterns which can be inferred by that very representation (by remapping it into the relevant sensory modalities) will be experienced. Eventually, the acquired representation meaningfully connects a behaviour (policy) with the sensory states that reliably follow from it.

#### (iii) Learning also depends on competence prediction

In addition to the convergence between internal representations, the learning process is also modulated by a statistic measuring how much a policy generated by an internal representation produces behaviours that consistently lead to high values of the learning signal. This statistic is collected cumulatively by a further component of the system, which learns to predict the probability of a given internal representation to produce a successful behaviour: competence predictor [58,59]. A high predicted probability thus informs about the competence of the agent, of its ability to reproduce those target sensory states whose representations are also representations of the current policies. These policies correspond to behaviours which are related to the progressive convergence between sensory and policy internal representations. The use of competence prediction in the learning process of the offline control layer is twofold. On the one hand, a mean value can be computed which informs about the overall competence acquired by the agent within the experienced environment, which is useful to modulate the overall learning and to avoid further learning when sufficient competence is reached. On the other hand, the competence of each policy can be computed at the beginning of each training episode, just after the policy is selected and the behaviour is chosen. In this way the learning step for the current episode is modulated and it is possible to avoid catastrophic interference in the learning process. This local competence statistic can also modulate the amount of noise to add to the selected policy so that exploration in the policy space is allowed.

#### (iv) Implementing the acquisition of topologies: supervised topological maps

A crucial assumption behind our hypothesis is that, to be able to align different modalities into a common internal space, an agent needs to control how the internal mappings of its sensory inputs and its motor programmes are developed. As anticipated before, an issue related to the development of a mapping between sensory states and motor commands that is faced by any agent in a realistic physical environment and with a high number of degrees of freedom is redundancy: there are usually many possible motor trajectories to obtain the same final state [43]. Following our hypothesis, if a lower-dimensional manifold can be found which is aligned between all sensory and motor spaces, then an agent can acquire an unambiguous mapping between sensory states and motor programmes. Finding such a lower-dimensional common manifold requires both (i) the ability to find a lower-dimensional topology for each of the original spaces, and (ii) the ability to constrain the choice of the proper manifold from each modality so that they are all aligned (a point in the manifold binds information on the same event in several sensory and motor modalities). We implemented this main feature by using an algorithm that allows us to learn a topological map from a stream of multidimensional input data in a controlled manner.

A classical method for finding the topological structure within a dataset of multidimensional patterns is the self-organizing map (SOM) algorithm [65]. This method produces a grid of prototypes of patterns in a given dataset in an unsupervised way. Within the emerging grid, each prototype is similar to a small group of patterns in the dataset. Moreover, each prototype is closely similar to its neighbours within the internal structure of the grid. Thus proximity between points in the grid recalls the similarity between input patterns in the dataset. The internal structure of the grid is predefined, such that, for instance, prototypes can be disposed in a lower-dimensional space. In this way, the multidimensional input data in the original dataset are reduced to a lower-dimensional representation (e.g. two dimensions in a simple case) that maintains the original topology, or, to put it in other words, a lower-dimensional manifold is found within the multidimensional space of the data. The learning process used to obtain the final topological structure is iterative. For each pattern of the input data, the following two steps are computed: (i) a prototype in the grid is chosen whose distance from the input is minimal with respect to other prototypes; and (ii) the chosen prototype and its neighbours are changed a little bit so that they get closer to the presented pattern. After each epoch (a complete iteration over all the patterns in the dataset) the neighbourhood is redefined so that prototypes must be closer in order to be considered as neighbours to each other, as far as epochs move on. It can be shown that this algorithm corresponds to the minimization of a weighted sum of the distances between all input patterns and all the prototypes in the SOM grid. Specifically:

2.1 $$L=\frac{1}{2}\sum_{i=1}^{N}\sum_{\;j=1}^{K}\phi_{i,j}\|x_{i}-c_{j}{\|}^{2}$$

with

2.2 $$\begin{array}{r} r_{i}=\underset{k}{\mathrm{arg min}}\|x_{i}-w_{k}\| \end{array}$$

and

2.3 $$\phi_{i,j}=\;e^{-(\|j-r_{i}{\|}^{2}/2\sigma^{2})},$$

where *N* is the number of input patterns, *K* is the number of prototypes in the output grid, **x**_*i*_ is the *i*-th input pattern, **c**_*j*_ is the *j*-th prototype, *r*_*i*_ is the index of the prototype at the minimal distance from the *i*-th input pattern and *ϕ*_*i*,*j*_ is the radial basis of the distance between the winning prototype for the *i*-th input pattern and the prototype *j* (see [66] for details). The SOM algorithm produces a lower-dimensional topological map within an internal representational space. Unfortunately, since the learning process is completely unsupervised, nothing can be said beforehand about the final positions of features within the acquired map.

However, to formalize our hypothesis, we need to be able to control the way in which mappings are acquired instead of leaving them free to develop in an unsupervised manner. In this way, different topological maps can be aligned while they develop, with the result that features within a region of space in a map correspond to features in the same region of another map.

This algorithm can be obtained by a generalization of the SOM algorithm, in which, during the learning process, the choice of the winning prototype depends not only on its distance from the input pattern but also on an external factor, for example the distance from a given position within the internal space where the manifold of prototypes lies down. In this case, the function to be minimized is the same as the one shown in equation (2.1) with the only difference that the weighting factor *ϕ* becomes a composition of a function of the distance from an input pattern (as before) and a function of the distance from an arbitrary given position *t* in the prototype grid (see [66] for details):

2.4 $$\phi_{i,j}=e^{-(\|j-r_{i}{\|}^{2}/2\sigma_{r}^{2})}\;e^{-(\|j-t_{i}{\|}^{2}/2\sigma_{t}^{2})}$$

We call such an algorithm ‘supervised topological maps’ (STM) because a label (a position in the internal space) is used to constrain the representation of each input pattern into a prototype lying in a controlled region within the internal grid [66]. Through this process, a topological map can be obtained whose topology is constrained so that some features are anchored in a region of the internal space. Given the STM method, two or more topological maps can be aligned together by constraining pairs (tuples) of patterns in two (or more) input modalities to a common anchor point in the internal space of the manifold. This algorithm for controlling the development of topologies is here adapted by using the representation of the currently generated policy as the anchor point in the internal space. In this way, all representations generated from sensory states encountered during the execution of a policy are confined to a region of the internal space near the representation of that policy.

A full description of the algorithm for STMs together with an introduction to SOMs can be found in [66].

#### (v) Implementing the intrinsic learning signal

Once the sensory states in each modality and the set of policies constituting the motor repertoire of the agent are mapped as points into the common internal space, it is easy to compute the linear distances between them. In particular, the intrinsic signal is computed at each timestep of an episode of a simulation (see §3).

At the beginning of each episode, a specific policy is selected. To allow for exploration, however, the policy that is actually executed in a given episode is defined by adding some random noise to the policy that was initially selected. Hence, the mean of the Euclidean distances between the points representing the current sensory state in each modality, the executed policy with the added noise, and the policy that was originally selected at the beginning of the episode can be computed as follows:

2.5 $${\hat{\xi }}_{t}=\frac{\sum_{i}^{N_{s}}\|x_{{s_{i}}_{t}}-{\hat{x}}_{\pi }\|+\|x_{\pi_{t}}-{\hat{x}}_{\pi }\|}{N_{s}+1},$$

where *t* is the current timestep, $x_{{s_{i}}_{t}}$ is the point that is mapping the sensory state *s* for the sensory modality *i* ∈ {1, 2, …, *N*_*s*_}, ${\hat{x}}_{\pi }$ is the point that is mapping the policy initially selected for the current episode and $x_{\pi_{t}}$ is the point that is mapping the policy with added random noise, which is the one actually used in the episode.

Once ${\hat{\xi }}_{t}$ is computed, we can transform it into a radial function to obtain a measure *ξ*_*t*_ whose maximum is 1 when ${\hat{\xi }}_{t}$ is 0, and is near 0 when ${\hat{\xi }}_{t}$ is highly negative or positive:

2.6 $$\xi_{t}=e^{-({{\hat{\xi }}_{t}}^{2}/2\sigma_{\xi })}.$$

The increment *δ*_*t*_ of *ξ*_*t*_ can then be easily computed as its change with respect to the previous timestep:

2.7 $$\delta_{t}=\xi_{t}-\xi_{t-1}.$$

Finally, we combine these two measures to define our intrinsic signal:

2.8 $$\psi_{t}=\left\{ \begin{array}{cc} \xi_{t} & \text{if}\;\delta_{t}>th_{\delta }\\ 0 & \mathrm{otherwise}. \end{array}\right.$$

High values of *ψ*—near 1 for instance—indicate that sensory and policy representations are converging, that their mean distances are very low, and that the representations match. If this convergence is statistically robust, the value of *ψ* is consistently high across situations, it can be assumed that the experienced sensory change is not accidental but an effect of the ongoing action, that a motor-sensory contingency has been detected. The signal can hence be used as a filtering mechanism to identify the experiences that are relevant for learning and thus to weight the tuples of sensory and policy states that participate to the update of the topological maps.

#### (vi) Implementing the competence prediction

The predictor component can be implemented as a logistic regression which learns to predict (the radial basis of) the distance between a representation of the policy and the representations of the sensory modalities experienced during an episode, that is the amount of intrinsic signal to be experienced when that policy is chosen. Thus the predictor component gives a statistic of the mean competence. This statistic of competence is used to modulate the main hyper-parameters in the learning of all supervised topological maps. This modulation is twofolded. On the one hand, the current competence is computed by the predictor at the start of a set of episodes (an epoch). To get an overall, *global competence*, the mean of all current competences for all the policy prototypes in the policy topological map is computed. The global competence signal is used as a global learning rate for all the topological maps for the current epoch. On the other hand, the competence of the single policy chosen at each episode is computed by the predictor. This *local competence* is used as a local learning rate for the further scaling of the topological update based on data collected during that particular episode. In this way, the learning of the topological maps is locally modulated. The learning rate based on global competence ensures that the maps are not changed anymore when the agent has acquired sufficiently high competence for all the points in its internal space of the policy representations. The learning rate based on local competence allows us to focus learning on those policies for which competence is still low (see also §3d; see [67] for a similar use of local modulation in SOMs). This twofolded mechanism for setting the learning rate allows us to avoid catastrophic forgetting when using algorithms such as SOMs or STMs with an online stream of data (lifelong learning, [68]). Finally, the local statistic about competence given by the predictor component is also used to arbitrate between exploration and exploitation in choosing the current policy. In particular, when the local competence for the current policy is close to 0 (low competence) a higher amount of noise is added to the policy so that new policies can be tried out. Instead, a high value (close to 1) in the local competence indicates that a given policy can be ‘exploited’ so that there is no further need of exploring and no additional noise is added.

### (b) The online control system: from policies to motor commands

As anticipated, it is important to identify the level of abstraction at which topological maps of motor programmes must be defined. As stated before, we choose *policies* instead of *motor commands* as the implemented level of abstraction. Within the described model, motor commands are defined as patterns of activation of the motor actuators, which trigger changes in the agent’s movements. In the case of our simulated environment (see below), this output from the actuator system consists of an array of values defining the current desired positions of the joints of the body (a two-dimensional arm with a gripper). Differently from mere motor commands, policies should be conceived instead as sensorimotor rules of behaviour specifying which motor command has to be triggered in the presence of a particular sensory state. Policies consist in a set of parameters which controls the responses of the actuator system, e.g. the weights of a neural network taking as inputs sensory states and giving motor commands (arrays of values defining actuators’ states) as outputs. In our simulations, the online controller is implemented as such a neural network, with its weights being chosen at the beginning of each episode by the offline controller (see §3).

## 3. The model and simulation

Going back to our motivating example of the infant spontaneously interacting with the rattle, to test our hypothesis, we focus on the formation of object concepts in the context of a manipulation task. To this aim, we implemented a simulated embodied agent endowed with a set of actuators allowing for manipulation, and which has access to information from relevant several sensory modalities, like somatosensory and proprioceptive modalities. Furthermore, a visual modality is added to show how the effect of topological alignment can also influence modalities that do not or only partially impact the intrinsic signal. Indeed, it can happen that a behaviour influences only some sensory modalities and not others; in the case of manipulation behaviours, for instance, it is usually the case that the visual system of the agent keeps track of the target object while its arm and hand engage in motor adjustments aimed at reaching the object and then grasping or pushing it. In this context, while changes in visual input during a manipulation episode are not mainly driven by arm and hand movements, changes in proprioceptive and somatosensory inputs are very probably owing to motor behaviour, given that manipulation is defined as ‘an agent’s control of its environment through selective contact’ [69, p. 4]. We show that also in this case all sensory modalities can be aligned by detecting motor-sensory contingencies, so that the ‘passive’ sensory modalities, once aligned, act as contextual tags within the converged sensorimotor representations: visual features are in fact used to select the relevant policy in each episode.

### (a) The simulated environment

**Figure 2.**
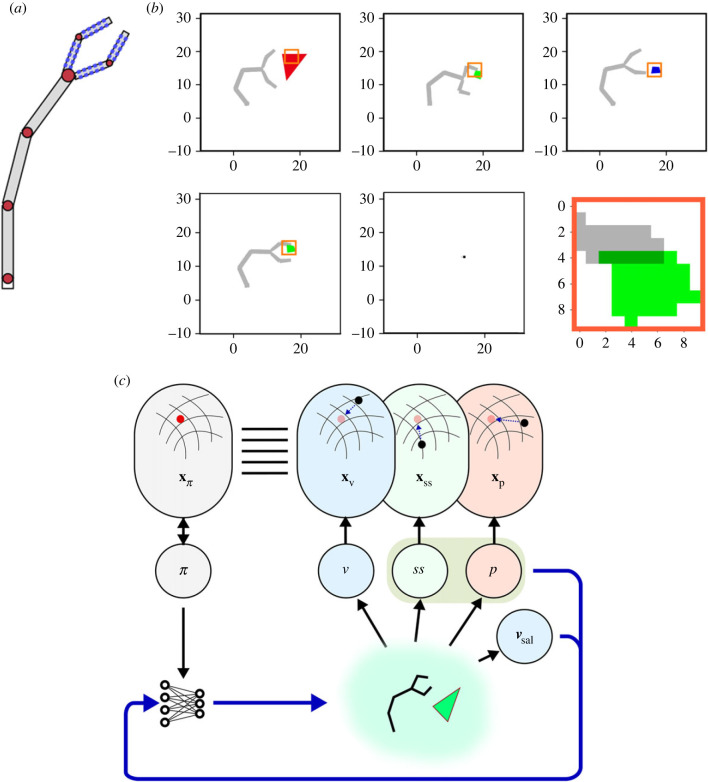
The agent and the simulated environment. (*a*) Schema of the agent. The 40 touch sensors (blue boxes) are spread uniformly over the internal and external sides of the gripper’s claws. Joints (red circles) are independent from each other with the exception of the two joints between the phalanges, which are coupled together. (*b*) Visualization of the environment: the agent and the possible contexts. In the top row, the agent is shown in three contexts where three different objects (red movable, green manipulable and blue fixed) are presented. In the bottom row, an example of the visual saliency (bottom centre) and retinal vision sensory states (bottom left, related to the green objects at the bottom right) is shown. (*c*) A schema of the two-layer architecture controlling the agent in the manipulation task. In the schema, ***π*** denotes the policy space, ***v*** the visual space, ***ss*** the somatosensory space, ***p*** the proprioceptive space, and ***v***_**sal**_ the visual saliency space. With the exception of ***v***_**sal**_, the sensory and motor spaces are mapped in their respective representation spaces, **x**_**π**_, **x**_**v**_, **x**_**ss**_ and **x**_**p**_ which become aligned over time. The blue arrow denotes the loop of the online controller. The black arrows denote the forward and inverse mapping between the offline and online control systems. (Online version in colour.)

The simulation setting consists of a two-dimensional environment with interactions defined by force dynamics. The environment contains the agent, a two-dimensional arm ending with a two-fingered gripper. The arm of the agent has 3 degrees of freedom (d.f.), it can be extended or retracted by modifying the state of three hinge joints. At the end of it, a gripper with two fingers can be opened or closed. Each finger has 2 d.f. (two hinge joints), but the behaviour of the two fingers is coupled together, so that the gripper has actually 2 d.f. overall (figure 2*a*). The agent can interact by touching, moving and grasping several kinds of objects which are placed in front of it, one at a time. As said before, the agent has access to information from four kinds of sensory modalities: (i) the current angular state of its joints (proprioception); (ii) the activation of 40 touch sensors that are evenly distributed between the inner and the outer parts of the two fingers of the gripper (figure 2*a*) (somatosensation or touch); (iii) a ‘retinal’ visual map defined by a picture taken at a local squared region around the most salient point in the visual picture of the whole environment (retinal vision) (figure 2*b* *top row*); and (iv) a ‘salience’ visual map which gives information about salient cues in the visual picture of the whole environment (saliency vision) (figure 2*b* *bottom row*). In each episode, an external object is placed in the scene. This object is randomly selected from three categories of objects that exist in this world: (i) ‘fixed’ objects, small quadrilaterals which can be reached and touched but not displaced; these objects are coloured with a randomly chosen shade of blue; (ii) ‘movable’ objects, big triangles which can be reached, touched and pushed but not grasped by the agent; these objects are coloured with a randomly chosen shade of red; and (iii) ‘graspable’ objects, small quadrilaterals which can be reached, touched, moved and grasped; these objects are coloured with a randomly chosen shade of green (figure 2*b*). We used these three colour categories to reduce the computational complexity of the qualitative features of the objects provided by the sensory modalities and to facilitate their identification, similarly to the role played by the ‘what pathway’ in humans [70,71].

Finally, for each episode, the arrays of sensory states, chosen policies, and corresponding representations at all timesteps are stored. The update of the topological maps happens after a run of 24 episodes (an epoch) is completed (see §3d).

### (b) The offline control system selects the policy

Inputs from the first three modalities (proprioception, touch and vision) is received by the offline control system which learns three sensory maps to be aligned with the map encoding the policy space (the policy parameters defining the motor programmes). This component uses its current policy map to select the policy that will be executed at each episode. The internal common space in which the outputs of all mappings are defined is two dimensional. This choice is made for computational convenience, with no bearing on our general hypothesis.

During training, the policy that is selected for each episode is the one whose internal representation overlaps with the representation of the initial visual state. This process is implemented by picking the point in the internal space of the offline controller which encodes the current initial visual state and then mapping it backwards to the policy space (action selection in an exogenous or stimulus-tied mode). Noise is then added to the policy based on the competence predicted for that policy. Thus, as explained before, the actual policy is not equivalent to the policy encoded by the representation selected in the internal space. This allows for the kind of exploration necessary to learn the internal topological space of the policies in the same way as it happens for the other sensory modalities.

### (c) The online control system specifies the motor commands

Visual saliency is accessed only by the online control system in its closed loop with the environment. The online controller receives as additional input information from the proprioceptive and somatosensory modalities and it returns motor commands in the form of desired postures (the joints angles) of the agent (figure 2*c*). In particular, the online controller completely determine its gripper’s joints but it controls its arm’s joints only partially. The main control of its arm’s movements is left to an hardwired controller which takes as its only sensory input the current point of maximal visual salience (which usually refers to the external object) and commands postures where the gripper is put nearby the object. This was done to separate the learning of manipulation behaviours from the acquisition of reaching behaviours, thus simplifying the analysis of learning.

There are two main reasons for this choice (for a review see [6]). First, it is clear that reaching and manipulation (by grasping or pushing) only partially share their set of motor actuators with each other. Second, reaching needs information about the spatial location of the target, and it can be conceived to result from computing the inverse kinematics given a desired spatial location. This computation mainly needs information linked to the spatial location of distal objects (in the current simulation this information can be reduced to the visual saliency map of the environment). Conversely, manipulation needs information about the discriminative features of an object. This information is mainly conveyed by the visual sensory state (foveal vision—reduced in our simulations to the retinal visual modality) as well as information from touch events (somatosensory information—implemented in the current simulation as information from the 40 touch sensors).

### (d) The learning algorithm

The pseudo-code in algorithm 1 describes the iterative algorithm used during training. The functions **visualMapping**, **touchMapping**, **proprioMapping** and **policyMapping** indicate the topological mappings from the sensory and policy spaces. All these functions give as their output both a simplified representation of the state in each modality as a single point in the inner space ($x_{v_{t}}$, $x_{ss_{t}}$, $x_{\;p_{t}}$, $x_{\pi_{k}}$, ${\hat{x}}_{\pi_{k}}$), and a full representation in the form of arrays of activations on a grid of points in the inner space ($r_{v_{t}}$, $r_{ss_{t}}$, $r_{\;p_{t}}$, $r_{\pi_{k}}$, ${\hat{r}}_{\pi_{k}}$) (see caption of algorithm 1 for the definitions of these symbols). Point representations are used in computing the intrinsic learning signal based on distances in the inner space, while grid representations can be used for inverse mapping (as in the function **PolicyInverseMapping**) from the inner space to a single modality. The grid representation of the chosen policy ${\hat{r}}_{\pi_{k}}$ is also used as the label for updating all topological maps in a supervised way (see §2a(iv)). The whole loop of iterations is first subdivided into epochs. During each epoch a number of episodes is simulated (number_of_episodes=24). At the beginning of each epoch, the mean competence $C_{\mathrm{tot}}$ for the grid of prototypes related to policy representations is computed. Then the iteration through the episodes begins. For each *k* episode, the initial sensory states are first read from the environment **env** and their current mapping into the internal space are computed. Then the current visual mapping is used to map backwards to the corresponding policy that becomes the policy selected ${\hat{\pi }}_{k}$ for the ongoing episode. To this selected policy, a noise ϵ is added, whose amount is modulated by its predicted competence *c*_*k*_. In this way, the policy that is actually used in the episode is defined ****π*_*k*_***. At this point, the iteration through the timesteps of each single episode begins. For each timestep, an iteration of the closed-loop control is computed, where the online controller **agent** gets the current sensory state and returns the current motor command, which is implemented in the environment resulting in the update of the sensory state. After the end of the iteration of the closed-loop control, the current internal representations in the offline control system are computed together with the current intrinsic learning signal *ψ*. All information about sensory states, the policies actually used in each episode, and distances in the inner space for each timestep is stored for all the episodes within an epoch. At the end of each epoch, both the topological maps and the competence predictor of the offline control system are updated based on this stored history. The topological maps update is based both on the intrinsic learning signal at each timestep Ψ and on the grid representations of the originally selected policy ${\hat{R}}_{\pi }$, which are used as labels to supervise the update of the topological maps (see §2(a)). Finally, the update is also inversely proportional to the current competence *c*_*k*_ for the policy representation ${\hat{r}}_{\pi_{k}}$ for each episode, so that episodes for which a high competence is predicted participate less to the update of the topological maps.

## 4. Results

With the aim of assessing the consistency of our hypothesis, we analyse a simulation of the process model in the manipulation setting. Results indicate that achieving a topological alignment between sensory and motor spaces is sufficient to endow an agent with a perception system that is stable and oriented towards the action possibilities that the environment affords to the agent. In this way, we provide evidence that our process model can indeed generate clusters of sensory prototypes that are discriminative of the relevant features of the environment and clusters of motor prototypes that can be used to select actions leading to those effects. Once an aligned sensorimotor system with these properties is available, we show that re-enacting the sensory and motor experiences starting from their internal representations can provide the basis to kick-start the formation of concepts.

### (a) Stabilization of topological maps via intrinsically-motivated learning

**Figure 3.**
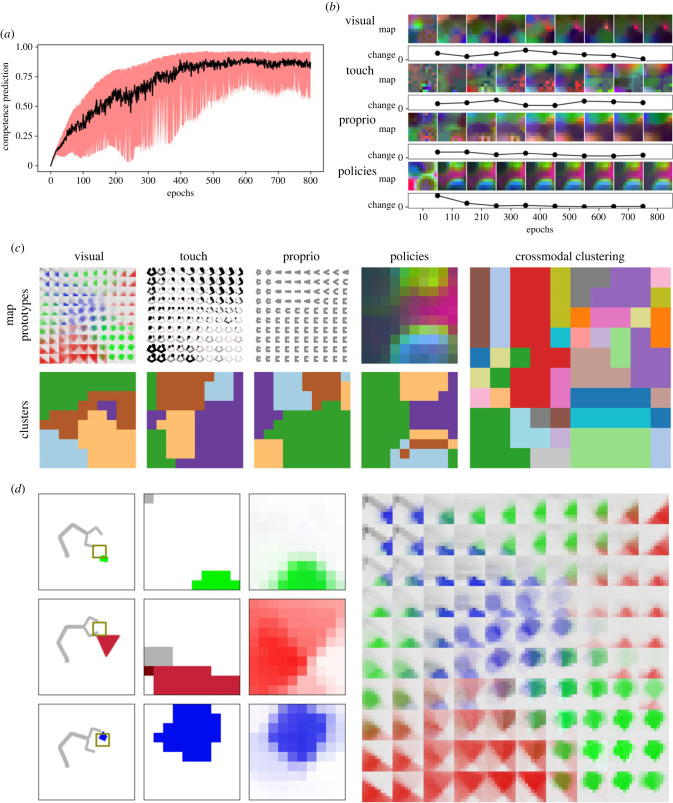
Analyses of equilibrium, stability and categorization system of the simulated model. (*a*) Curve of the mean competence through epochs. (*b*) Structure of the topological maps throughout the training phase, with values of Euclidean distances between maps at different epochs. (*c*) Visualizations of the prototypes in the four topological maps after learning along with a cluster analysis for each map and the cross-modal clustering. (*d*) Re-enactment of sensory states in different contexts. (Online version in colour.)

In order to analyse the sensorimotor representation system that emerges from the interactive experiences of the agent, we need first to check whether learning has reached an equilibrium. The curve of global competence through the learning epochs can be used to ascertain the learning equilibrium. As described in §3d, global competence is computed as the mean of competence predictions for the grid of policies in the policy map. This is a measure of how much the agent is able to induce the sensory effects that are encoded in the same internal representation which is also encoding the current chosen policy. Figure 3*a* shows that this curve increases over time and reaches a steady equilibrium. When the environment has been sufficiently explored in its various contexts (the different objects), the agent becomes highly competent for all events included in the setting. The amount of changes in the maps over time can instead be used to test that learning has led to stable aligned topologies. At equilibrium, no changes should be recorded anymore in the topological maps since they correspond to a learned stable manifold in the sensory and policy spaces. An analysis of this rate of change in the development of the topological maps is presented in figure 3*b*. In each of the four subfigures (visual, touch, proprio and policies), the first epochs of development of each of the four maps is shown. For each epoch, a 10 × 10 colour matrix is shown in which each pixel defines a prototype projected into the internal space. Since the dimensionality of the four spaces differ (e.g. the visual space consists of 10 × 10 × 3 pixels while the policy space consists of 500 parameters), all prototype datasets were reduced through a principal component analysis through their first three components. Thus each prototype was reduced to a three-dimensional array which can be represented with a red-green-blue (RGB) colour. Each subfigure shows at the top the representation of the corresponding topological map at the first epochs of training. At the bottom of each subfigure, the Euclidean distance between the same map at various time intervals is shown. As it can be seen, this measure of change stably reaches values near zero at the end of the time series for all the modalities. The touch modality, where this measure is less stable, seems to be an exception. This effect can be ascribed to the fact that events in which the agent actually touches the object are only a subset of all events in which a high value of the intrinsic learning signal is found. Thus the dataset of touch patterns that is used to learn the topological map is smaller than those of the other two sensory modalities.

### (b) Development of a cross-modal and action-oriented categorization system

Once the topological maps are steadily formed, the prototypes of their output activations are representations of sensory states and motor programmes organized in a two-dimensional grid in the internal space. These prototypes also are aligned across modalities, and with the external environment, which means that a tuple of the prototypes from each of the four modalities (vision, touch, proprioception and policies) on the same node of the grid binds these pieces of information into the prototype of a motor-sensory contingency or action-effect association. Figure 3*c* shows the prototypes obtained for each modality after learning. In particular, the four plots on the top left show the prototypes as sensory states generalized over all training experience. In the first plot from the left (visual), each square in the grid is a prototype of retinal states. The second plot (touch) depicts the maps of somatosensory states. In this case, each prototype in the top grid is drawn as a sketch of the gripper. In each of these sketches, the activation of touch sensors is represented as the radius of black dots centred on each of the 40 sensors over the gripper. The third plot (proprio) visualizes the proprioceptive map. As before, each prototype in the top grid is drawn as a sketch of the gripper. Here, however, the proprioceptive states are shown as the current angular position of the two pairs of joints of the gripper. The last column on the right of figure 3*c* shows the policy map. In this case, each prototype in the grid cannot be easily graphically represented because each state is a high-dimensional array of the parameters which defines a policy. Thus a dimensionality reduction was computed on the prototypes, and the first three components are represented in the grid as RGB colours. By inspection, it is clear that, after learning, each modality is segmented into well-defined regions of the topological space. From each of the four topological maps, a clustering of the prototypes is also visualized (bottom-left plots). Clustering was produced through a *k*-means procedure with *k* = 5. Clusters can be easily recovered in the visualization of the prototypes in the plots at the top. Note that clustering takes into account that multidimensional information is reduced to a two-dimensional representation. For instance, in the visual modality, it can be easily seen that clusters actually include both colour and shape information. Finally, the plot at the right of the figure is a linear composition of the four clusters at the bottom left. Thus each cluster in this cross-modal elaboration visualizes a group of points in the internal space which have similar prototypes to each other in all modalities. We suggest that these clusters can be viewed as smooth categories of motor-sensory contingencies.

### (c) Concept formation as offline multimodal simulations

Starting from the cross-modal categorization system, the offline control system has the resources to *endogenously re-enact* the sensorimotor states related to an internal representation, in a way that is consistent with the grounded approach to concepts [9,11]. A representation in the topologically aligned internal space can be used endogenously to generate a prediction of the multimodal sensory inputs to be experienced as a consequence of taking a particular action (forward model). When the *same* representation in the internal space is used to re-enact a motor programme in the policy space (inverse model), an action as a meaningful goal-directed movement is realized. This sensorimotor re-enactment is shown in figure 3*d*, using the visual modality as an example of what it is like for the agent to re-enact a pattern in a particular modality starting from an internal representation. In particular, figure 3*a*–*c* shows three offline simulations in three different contexts (green graspable objects, red movable objects and blue fixed objects). Each row shows both the agent’s current state (left), what the agent is currently viewing in its retina (centre) and what the agent is currently re-enacting in the visual space (right). Figure 3*d* shows a complete grid-view of the prototypes in the visual topological map when learning has reached equilibrium. The same re-enactment of sensory states starting from their internal representation is possible for the other modalities too. Note that this test also illustrates how an internal representation can be used as a *goal*, in which the agent is presented with a context and the representation of the very first view of the object at the start of the episode is used to select and re-enact a policy which is then carried out in the rest of the episode.

### (d) Perception-action integration in the internal convergence dynamics

If topological alignment results in the integration of perception and action (as in [39]), the perception of an external event should be sufficient to prime the action system to select a behaviour aiming to reproduce the target sensorimotor state that has been learned in that context, its *goal*. One way to check if this integration has been achieved is to explore the internal dynamics of the agent’s internal sensory representations during an episode. This representation dynamics is affected by the continuous stream of sensory inputs during the episode and it should converge towards the region in the common representation space that is defined by the selected policy. The dynamics of the internal representations during behaviour can be viewed as moving towards an attractor, which is in fact the goal that the agent is pursuing.

**Figure 4.**
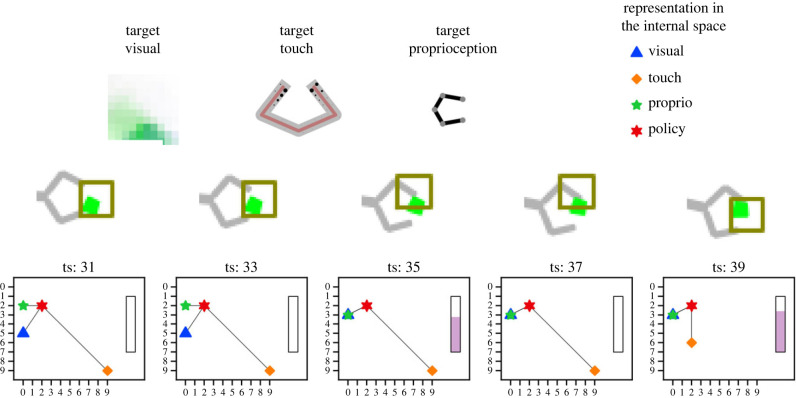
Representation of the convergence of representations of different modalities throughout an episode. At the top left, the three target sensory states generated via a backward mapping from the internal representation of the initial policy (re-enactment). At the bottom, a sequence of time-steps leading to a convergence event after learning, where the motor-sensory contingency is reproduced and the target state is re-experienced. For each timestep, the current snapshot of both the agent and the environment is given as well as the position of the representations of all four modalities in the internal space. Black lines are added to visualize distances between sensory representations and the representation of the policy. The vertical bar at the right side of the representation plot for each timestep indicates the amount of intrinsic signal *ψ* which is related the current change in the mean of the four distances. (Online version in colour.)

An illustrative example of this internal dynamics is provided in figure 4. The figure describes the internal dynamics of representations during an interval of timesteps within an episode. The interval has been chosen so that the intrinsic signal *ψ*, which describes a change in the mean distance between representations, increases in the final timestep. The three plots at the top-left of figure 4 show the sensory states in each of the three modalities (visual, touch and proprioception) as they are generated via a backward mapping from the policy representation (re-enactment). These re-enacted states also describe a state which minimizes the distance between the sensory and the policy representations in the internal space. The sequence at the bottom of figure 4 is composed of pairs. For each pair, the graph at the top is a snapshot of the current position of the agent and the current object, with a red square indicating the current retinal field of view. Directly below, the sequence shows the point-representations for each modality in the internal space. Black lines visualize the distance of current sensory representations (the blue, orange and green symbols) from the policy representation (the red symbol). The bar at the right in the plot indicates the current value of the signal *ψ*. This sequence shows how representations change their position and their distance from the policy representation so that they get closer to it in the final timestep. It is easy to see how, in the given example, representations begin to converge first in timestep 35, when the visual inputs become similar to the target. Then an increasing convergence signal is triggered at timestep 39 when the agent actually touches the object with a pattern of activation that is even more similar to the target touch allowing also for the internal somatosensory representation to converge with the policy representation. Thus the analysis of the internal dynamics of representations during behaviour once the topological alignment is acquired shows that the agent is able to select the policy that will reproduce the motor-sensory contingency relevant for the current context.

**Algorithm 1.**
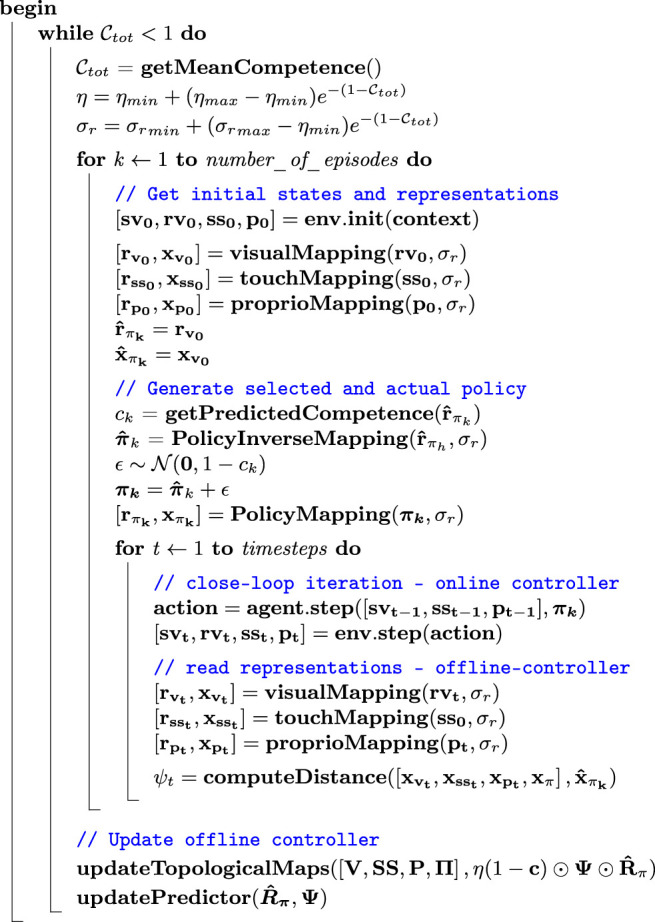
Pseudocode describing the learning algorithm. $C_{\mathrm{tot}}$: mean of competence values collected over a grid of points in the inner space; k: index of current episode; t: current timestep; η: learning rate of mappings update; $\eta_{\min}$ minimum eta value (when grid competence $C_{\mathrm{tot}}$ is equal to one); $\eta_{\max}$ maximum value (when grid competence $C_{\mathrm{tot}}$ is equal to zero); sv: saliency vision state; rv: retinal vision state; ss: touch (somatosensory) state; p: proprioceptive state; $\pi_{k}$ policy actually used in episode k; ${\hat{\pi }}_{k}$ policy selected for episode k before noise is added; ϵ white noise added to the chosen policy; $r_{v}$ grid representation of the visual state; $r_{ss}$ grid representation of the touch state; $r_{p}$ grid representation of the proprioceptive state; $r_{\pi }$ grid representation of the policy; ${\hat{r}}_{\pi }$ grid representation of the selected policy before noise is added; $x_{v}$ point representation of the visual state; $x_{ss}$ point representation of the touch state; $x_{p}$ grid representation of the proprioceptive state; $x_{\pi }$ point representation of the policy; ${\hat{x}}_{\pi }$ point representation of the policy selected before noise is added; $c_{k}$ current competence for the selected policy ${\hat{\pi }}_{k}$; $c_{k}$ current competence for the selected policy; $\psi_{t}$: intrinsic learning signal computed for timestep *t* V: array of visual states for all timesteps in all episodes of an epoch; SS: array of touch states for all timesteps in all episodes of an epoch; P: array of proprioceptive states for all timesteps in all episodes of an epoch; Π: array of policies for all episodes of an epoch; c: array of predicted competences for all episodes of an epoch; Ψ: array of intrinsic learning signals for all timesteps in all episodes of an epoch; The operator ⊙ stands for the elementwise product between arrays.

### (e) Semantic content in the internal representation space

However, how can we establish that these internal representations have meaning in the sense of being grounded in the external environment? Also, how can we fix the semantic content of a representation in the internal space?

We have now the resources to provide an answer to this question. We start by noticing that the internal dynamics of convergence between representations is in fact also a measure of the agent’s *success* in achieving her goal. Similarly to the action-based view of grounding, the content of a representation is determined by the conditions in which the action guided by the representation is successful, the success conditions of the action [34], which, in our approach, correspond to the convergence conditions of the relevant internal dynamics. Therefore, we propose that a point **x** in the topologically aligned internal space refers to—is grounded in—condition **C** in the environment if and only if the sensory representations **x**_**v**_, **x**_**ss**_, **x**_**p**_ activated in the internal space would converge with the representation of the policy ${\hat{x}}_{\pi }$ that is being used to control the closed-loop interaction with the environment *were **C** to obtain in the environment*. Whatever condition **C** in the environment is necessary for the internal convergence of sensory representations towards point **x** in the internal space, that is the condition **x** refers to, its semantic content.

Consider again the convergence dynamics of the example in figure 4. Here, the convergence between **x**_**v**_, **x**_**ss**_, **x**_**p**_ with ${\hat{x}}_{\pi }$ presupposes the kind of motor-sensory contingency that is afforded by the green object, which is thus part of the conditions that ensures internal convergence, i.e. successful goal achievement. In this view, the cross-modal region of point representations in the internal space whose convergence dynamics presupposes that presence of a green object is therefore its conceptual representation. Similarly to the rattle in Piaget’s observations, and in line with other computational proposals in this constructivist spirit (e.g. [26,28]), an object is conceived in virtue of the set of its expected interactions. However, crucially for our purposes, by defining success as internal convergence, no appeal to further semantic notions has been necessary.

## 5. Discussion

In this study, we have proposed an algorithmic-level account—a process model—of how concepts can develop when interacting in high-dimensional continuous sensorimotor domains. To demonstrate that this model has sufficient resources to address the kick-starting problem of how meaningful concepts form from meaningless sensorimotor streams of data, the model has been specified in a computational architecture and tested in a (simulated) manipulation task. We have proposed that meaningful representations arise if the control system of the agent is already equipped with an internal representation space since a common metric distance is needed to compare representations encoding the motor programmes of an agent with those encoding its sensory inputs even if, at the start, these representations are actually meaningless. Thanks to this comparison, the convergence over time between motor and sensory representations can be computed. Importantly, convergence between representations in the internal space is influenced by the actions that are actually taken in the environment and the subsequent changes in sensory inputs. Thus a convergence signal can be used to detect motor-sensory contingencies or action-effect associations in the high-dimensional continuous stream of sensorimotor experience. Given that the signal of convergence is entirely defined in the representational space and is used as a reward to guide learning of the representational space itself, it can thus be interpreted as reflecting an underlying intrinsic motivation for competence acquisition, a motivation to become able to effectively interact with the environment [52].

Results of an *in silico* experiment in an object manipulation task have provided evidence that even when an agent begins its life in an experiential ‘blooming, buzzing confusion’, it can learn to control its initially random behaviour in a goal-directed way (intentional action) while at the same time developing a behaviourally relevant categorization system that supports meaningful perception. Interestingly, the proper alignment of the categories in the policy map with those in the visual map makes it possible to select and specify the action, not only in an exogenous or stimulus-tied mode but also endogenously, by activating a goal in the internal space. In this way, the agent naturally learns to perceive the objects in terms of the action possibilities they afford and object perception can also bias action selection [39,72]. Moreover, our results demonstrate that the agent has also the resources to ‘detach’ [73] or disengage from its perceptual contact with the environment, to form rudimentary but meaningful concepts by re-enacting or endogenously generating its own multi-modal experiences [9,11], in a way that make this information potentially available to other cognitive processes.

Finally, our approach vindicates the action-based view of grounding by showing with a formal, computational model that its (pragmatist) logic can work without the need for assuming other meaningful or intentional notions along the way. An explanation that focuses on the *intrinsic* rewards of success can help us ‘break into the intentional circle’ [74, p.66]. Still, despite acquiring semantic content, representations formed by topological alignment do not encode action-invariant descriptions of objects as classically assumed, but preserve an essential ‘directive’ nature as well as their value for the task of controlling the state of the organism in the environment [7,75].

The grounding by competence acquisition hypothesis may also clarify why representations in the common internal space refer to distal features in the environment and not to their proximal causes in the sensory and policy spaces. As Wolfgang Prinz has nicely put it: ‘common coding has been the problem and distal representation is the answer, not vice versa’ [76, p. 18]. We agree. However, according to TEC, a common coding of action and perception refers to distal features because a common format can be found at the distal but not at the more proximal level of the sensory modalities or motor patterns [39]. Consider once again the encounter between the infant and the rattle. A successful grasping of the rattle requires that several features of the perceptual input and the action goal match, the intended grip, for instance, should match the perceived size of the object. While this is true, our approach and model clarify instead how a match (or convergence) in the common representation space is possible even when representations are meaningless at first, and that content gets fixed only once the alignment process is completed. While distal reference is semantic content, proximal reference is just a ‘code’ (see [22]).

The computational model and its implementation has been used to provide a proof-of-principle of a mechanism for concept formation starting from spontaneous exploration of objects. More specifically, we have shown that a measure of the amount of alignment of sensory and motor policy topological maps is in principle sufficient to guide the intrinsic learning of sensorimotor contingencies and, on this premise, to kick-start cognitive development. This view resonates with the Piagetian approach to the origin of human intelligence [3] and contributes by providing a novel, missing algorithmic-level explanation that can bridge the gap between developmental psychology and dominant embodied cognition approaches, which are still mostly focused on adults [77]. As for other cognitive skills and functions [49,55], here we have demonstrated that an intrinsically motivated learning process can also support concept formation.

From an evolutionary standpoint, it has been suggested that the cognitive mechanisms for predicting action-effects (forward models) may have first evolved to support online action control in complex dynamic environments (e.g. compensating for delay or noise in sensory signals), and that they may have later become available to enable more complex forms of goal-directed or intentional control, which, by inverting the process, use these predictions to select the actions that will cause the predicted sensory effects [30,36,73]. Here, adopting an ontogenetic perspective, we may instead suggest that it might be useful (at least developmentally) to explore the other way: the acquisition of offline (goal-directed) action control in simpler and less noisy environmental conditions that do not require predictive control strategies may create the representational means to generate the sensory predictions endogenously in the format that online action control will be able to exploit in more dynamic environments. This strategy may reduce the computational costs of learning to predict directly in the raw space of sensations, which is in fact a daunting task [78].

While a detailed comparison with alternative models of concept learning is beyond the scope of this contribution (e.g. [79,80]), it is important to note that most of the leading computational approaches are focused on adult human performance, avoid the question of how sensory and motor information is encoded, and assume that a meaningful sensorimotor system is already available (for a review see [81]). Here, we have taken a step to overcome these limitations.

In addition to this psychological plausibility, the main features of our architecture are consistent with key principles of brain organization. First of all, topological organization is ubiquitously implemented in the brains of vertebrates. Functional topologies over the features of sensory information can be found at the cortical level as in the organization of the cortical columns of primary and associative sensory areas [82,83]. Furthermore, a topology based on motor programmes is also present in the motor cortical areas [84–86]. Topological maps align with each other into multimodal networks, for instance, to route copies of motor commands (corollary discharges) to relevant sensory structures [87]. Importantly, there is evidence that at least some of these multimodal alignments are acquired in an activity-independent manner, as it happens in the superior colliculus, where visual, somatosensory and auditory modalities are integrated [88]. This last evidence, in particular, is in line with our main assumption that different modalities must share a common metric at the level of the internal space of their representations so that a representation in one modality can be compared with a representation in another modality. More generally, our approach is compatible with an emerging perspective in neuroscience according to which human concepts in the brain are organized in lower-dimensional spaces recruiting structures originally evolved for interaction with the physical world, e.g. for spatial navigation [89,90]. Here, we have shown that an analogous strategy may also hold for the organization of less abstract conceptual knowledge: grounded knowledge that derives from physical manipulation and preserves a tighter connection with its sensorimotor origins.

Our process model has been designed with the aim to capture the minimal system needed to acquire a meaningful sensorimotor representation system. However, several limitations of its current implementation can be identified.

For instance, to ease its computational tractability, we have not constrained the initial motor repertoire of our agent even if bodily movements of most animals (humans included, [91]) result from a set of initial motor primitives and biophysical forces that orient motor development. However, no assumption about the initial configuration of the space of policy parameters (our motor or policy space) is needed by our model and adding a specific set of motor primitives, for instance, would provide additional constraints to the generation of the internal sensorimotor manifold while still being consistent with our hypothesis. Indeed, starting from motor primitives and learning an internal manifold in the policy space are two complementary ways for a motor system to take advantage from its abundance of degrees of freedom, thereby bypassing the issue of redundancy [92].

A further limitation of the current implementation is that, despite the topological alignment of the different modalities in the internal space, their multisensory or multimodal integration play only a minimal role. For instance, while the policy of an episode is selected starting from the visual modality (by picking the overlapping point in the common space), to simplify learning, this interaction is defined in an *ad hoc* manner at the start of each learning episode. However, direct interaction among the different sensory modalities and their mutual influence during online interaction is an important extension, which would adapt the model to study, for instance, fully ‘embodied’ choices in which the processes of action selection and action specification proceed in parallel [72,93,94].

Lastly, another relevant limitation is owing to the deterministic selection of the internal representation from which the policy of a given learning episode is generated. Unfortunately, this way of selecting the ‘goal’ to be pursued in the episode does not allow for an exhaustive search of all the policies that might be relevant for a given context, thereby running the risk of finding a suboptimal manifold for the policy space. The standard approach to deal with this problem is to add some stochasticity in the process of goal selection. However, beside a purely random approach, an intrinsic motivation like competence acquisition may also be employed so that the model would indeed acquire further a capacity for curiosity-driven goal exploration which is now missing [57–59]).

Addressing these limitations will also be important to meet a recurrent objection to the grounded approach to cognition, according to which its plausibility is drastically reduced when attempting to explain the representation of abstract categories for which sensory and motor experiences are only relevant to a very limited extent, if at all [95]. Besides the crucial role of language development to ‘unground’ concepts and create abstract symbols [96], one way to address this challenge is to consider that beside the domains of action and perception tied with interaction with the outside world, the grounded approach to cognition assumes that conceptual representations can also be grounded in introspective experiences of *one’s own mind*, via higher-order *metacognitive* signals representing, monitoring and controlling lower-order cognitive processes [9,11]. On a par with exteroceptive and interoceptive experiences, these metacognitive experiences have been considered as a source of grounded knowledge, but one that is especially important for more abstract (as opposed to more concrete) concepts like those of truth, falsity, freedom and the like [97,98]. While often acknowledged, this possibility has had a very limited impact in the experimental literature so far, probably owing to the lack of rigorous process models to clarify it.

Consider for instance the case of ownership of property: the abstract knowledge that an object is mine or someone else’s. Since ownership is invisible, it is often considered a prototypical abstract conceptual domain that apparently resists an embodied explanation [99]. However, knowledge that an object belongs to oneself may be grounded in the sense of possession, of how controllable by the self an object is [100] and an early understanding may actually develop even in prelinguistic infants as a by-product of their intrinsic motivation to explore, manipulate and control our immediate environment. It has in fact been proposed that, because of such intrinsic motivation, during their first 2 years of life, infants are under pressure to identify the objects in their environment that give rise to feelings of efficacy and personal control, and to keep them apart from those that instead thwart such feelings owing to the interference from other people [101]. The former class of controllable objects becomes the category of objects that are understood as belonging to the self, while the latter class includes those that are not. Casting this proposal with the resources provided by our process model might reveal that such a curiosity-based exploration relies on monitoring one’s competence improvement (or lack thereof), which is indeed a fundamental metacognitive signal [102].

Perhaps, a process model that explains even how these (early) abstract conceptualizations may form on top of simpler sensorimotor processes is within reach.

## Data Availability

Model code and data to reproduce the results reported in this manuscript are publicly accessible at https://doi.org/10.5281/zenodo.7437296.
